# Supplementary material for: The Evolution and Characterization of the RNA Interference Pathways in Lophotrochozoa
Source: Genome Biol Evol. 2024 May 7;16(5):evae098. doi: 10.1093/gbe/evae098 (PMC11114477; doi:10.1093/gbe/evae098)
Supplement: evae098_Supplementary_Data [file evae098_supplementary_data.zip › Supplementary_materials.zip!/selected_miRNAs/NoGe_selected_miRNAs/novel_miRNA_families_pdf_files/NMRB01000991.1_Notospermus_geniculatus_strain_Ushimado_scaffold991,_whole_genome_shotgun_sequence_23697.pdf]

Provisional ID	:	NMRB01000991.1_Notospermus_geniculatus_strain_Ushimado_scaffold991,_whole_genome_shotgun_sequence_23697
Score total	:	482.2
Score for star read(s)	:	3.9
Score for read counts	:	477.3
Score for mfe	:	1.5
Score for randfold	:	0
Score for cons. seed	:	-0.6
Total read count	:	948
Mature read count	:	921
Loop read count	:	0
Star read count	:	27

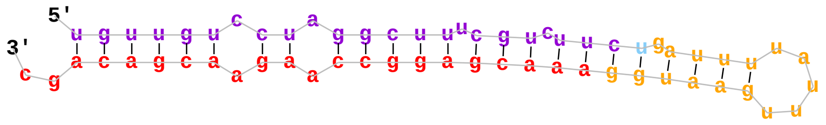

Genome coordinates: NMRB01000991.1 115830-115892

pre-miRNA sequence:
TGTTGTCCTAGGCTTTCGTCTTCTGTTGTCCTAGGCTTTCGTCTTCTGATTTTATTTGAATGGAAACGAGGCCAAGAACGACAGC

Mature sequence: AAACGAGGCCAAGAACGACAGC

BLAST results for the pre-miRNA sequence against the *Lineus longissimus* genome assembly:

Scaffold	Start	Stop	Identity	e-value
OU343004.1 1	3392306	3392246	95.082	8.48e-20

```
Nge_novel_miRNA        TGTTGTCCTAGGCTTTCGTCTTCTGATTTTATTTGAATGGAAACGAGGCCAAGAACGACAG
Llo_genome             TGTTGTCCGAGGCTTTCGTCTTCTGATATTGTTTGAATGGAAACGAGGCCAAGAACGACAG
```
